# Antiadhesive Properties of *Abelmoschus esculentus* (Okra) Immature Fruit Extract against *Helicobacter pylori* Adhesion

**DOI:** 10.1371/journal.pone.0084836

**Published:** 2014-01-09

**Authors:** Jutta Messing, Christian Thöle, Michael Niehues, Anna Shevtsova, Erik Glocker, Thomas Borén, Andreas Hensel

**Affiliations:** 1 University of Münster, Institute of Pharmaceutical Biology and Phytochemistry, Münster, Germany; 2 Umeå University, Medical Biochemistry and Biophysics, Umeå, Sweden; 3 University Hospital Freiburg, Reference Centre for Helicobacter pylori, Department of Medical Microbiology and Hygiene, Freiburg, Germany; Charité-University Medicine Berlin, Germany

## Abstract

**Background:**

Traditional Asian and African medicine use immature okra fruits (*Abelmoschus esculentus*) as mucilaginous food to combat gastritis. Its effectiveness is due to polysaccharides that inhibit the adhesion of *Helicobacter pylori* to stomach tissue. The present study investigates the antiadhesive effect in mechanistic detail.

**Methodology:**

A standardized aqueous fresh extract (Okra FE) from immature okra fruits was used for a quantitative *in vitro* adhesion assay with FITC-labled *H. pylori* J99, 2 clinical isolates, AGS cells, and fluorescence-activated cell sorting. Bacterial adhesins affected by FE were pinpointed using a dot-blot overlay assay with immobilized Lewis^b^, sialyl-Lewis^a^, H-1, laminin, and fibronectin. ^125^I-radiolabeled Okra FE polymer served for binding studies to different *H. pylori* strains and interaction experiments with BabA and SabA. Iron nanoparticles with different coatings were used to investigate the influence of the charge-dependence of an interaction on the *H. pylori* surface.

**Principal findings:**

Okra FE dose-dependently (0.2 to 2 mg/mL) inhibited *H. pylori* binding to AGS cells. FE inhibited the adhesive binding of membrane proteins BabA, SabA, and HpA to its specific ligands. Radiolabeled compounds from FE bound non-specifically to different strains of *H. pylori*, as well as to BabA/SabA deficient mutants, indicating an interaction with a still-unknown membrane structure in the vicinity of the adhesins. The binding depended on the charge of the inhibitors. Okra FE did not lead to subsequent feedback regulation or increased expression of adhesins or virulence factors.

**Conclusion:**

Non-specific interactions between high molecular compounds from okra fruits and the *H. pylori* surface lead to strong antiadhesive effects.

## Introduction

The immature fruits of *Abelmoschus esculentus* (L.) Moench, Malvaceae, also known as okra or lady's finger, are widely used as a food vegetable in Asia, Africa, and South-America. Because of its high amount of mucilage, okra is also used in traditional medicine as a dietary meal to treat gastric irritation. The structural elements of mucilage have recently been characterized as pectin-like rhamnogalacturonans [1], [3] with unusual structural features [2]. Additionally, these okra rhamnogalacturonan polysaccharides reportedly possess antiadhesive properties that interrupt the adhesion of *Helicobacter pylori* to human stomach tissue [3]. The antiadhesive effects are caused by an interaction with the bacteria, while pretreatment of human stomach tissue with the plant polysaccharides prior to the addition of *H. pylori* does not lead to reduced bacterial adhesion [3]. Polysaccharides have no direct cytotoxic effects against *H. pylori* [3].

Preparations with antiadhesive properties against pathogens may be interesting tools for future medical developments, as they interact with the surface proteins of pathogens that have not yet been pinpointed as molecular targets. Antiadhesive preparations cannot cure an acute infection, but might be used after eradication therapy to inhibit recurrence by preventing recolonization of the human stomach. Historically, the identification of antiadhesive compounds against *H. pylori* has been based on the initial finding of antiadhesive properties of 3′-sialyllactose [4]; unfortunately, this compound failed to prevent bacterial colonizaton of human stomach in a preliminary clinical study [5], likely owing to degradation of the compound under physiological conditions in the stomach. The search for additional antiadhesive compounds has yielded peptides [6], polyphenols [7]–[8], *N*-phenylpropenoyl-L-amino acid amides [9], and polysaccharides [10]–[11] that interact with bacterial outer membrane proteins (OMPs). The clinical and economic development of such antiadhesives is still underrepresented, in many cases because it is economically difficult to obtain sufficient amounts of these natural products at reasonable prices. Therefore, there is still a long way to go towards the development of registered antiadhesive drug products.

Recognition of and adhesion to epithelial cells by *H. pylori*, as well as the persistence of *H. pylori* in the stomach, is mainly mediated by several OMPs from the *Helicobacter* outer membrane protein (*hop*) group. Intensively studied members of the *hop* group include the blood group antigen binding adhesin (BabA, also known as HopS) and the sialic acid binding adhesin (SabA, also known as HopP). BabA interacts with fucosylated oligosaccharide structures present in H-1 and Lewis^b^ (Le^b^) blood group antigens [12]–[13]. BabA is also centrally involved in *H. pylori* binding to MUC5AC and MUC5B, even in non-secreting individuals that either lack an α(1,2)-fucosyltransferase (and are therefore not able to express Le^b^ in high amounts) or lack Le^b^, and thereby acts as an important factor for initial colonization [14].

Furthermore, antigens such as sialyl-Lewis^a^ and sialyl-Lewis^x^, which are predominantly expressed in inflamed gastric tissue, interact with and bind to SabA [15]. Such fucosylated and sialylated antigens favor the colonization of *H. pylori* to the gastric mucosa, and might even promote the chronicity of infection once gastritis is established [15]. SabA is also the hemagglutinin responsible for sialic acid-dependent hemagglutination [16].

The adherence-associated lipoproteins (AlpA and AlpB, also known as HopB and HopC), outer inflammatory protein (OipA, also known as HopH), and HopZ are also associated with bacterial adhesion [17]–[19], [23]. However, corresponding receptors for AlpA/B, OipA, and HopZ have not yet been identified. Another bacterial adhesin known as HpaA, a subunit of N-acetylneuraminyllactose-binding fibrillar hemagglutinin, can be blocked by the glycoprotein fetuin and 3′-sialyllactose. Exogenous 3′-sialyllactose can even reverse hemagglutination, and can detach adherent *H. pylori* from gastric cells [17]. In addition, interactions between *H. pylori* and extracellular matrix proteins such as laminin, fibronectin, and type IV collagen have been described to function as receptors in the gastric region [20]–[22].

With regard to these highly complex interactions and the importance of *H. pylori* adherence for the development of its pathogenicity, a precise comprehension of an inhibition mechanism for new antiadhesive compounds is essential. The life-long eradication of *H. pylori* by antibiotics is becoming increasingly problematic because of increasing antibiotic resistance, and the development of a prophylaxis by vaccination is challenging and still in progress [37]. Antiadhesive compounds could provide a preventive, cytoprotective strategy to control *H. pylori* colonization, especially to prevent its recurrence after antibiotic eradication therapy.

Several virulence factors have been elucidated to mediate pathogenicity and disease outcome of *H. pylori* infection. Aside from the flagella (which is important for mobility) and urease (which is necessary for acid tolerance), adhesins also play an important role: in addition to maintaining *H. pylori* colonization and persistence, adhesins also direct progression of the infection and incidence of the disease. Other virulence factors include the cag pathogenicity island (cagPAI) and vacuolating cytotoxin (VacA). Despite all knowledge about *H. pylori* attachment to the gastric epithelium, the associations and interactions between individual virulence factors and adhesion remain controversial. Therefore, we studied the hypothetical interaction of Okra fresh extract (Okra FE) as an antiadhesive agent and its influence on the mRNA expression of several OMPs, as well as virulence factor encoding genes *ureA*, *ureI*, *fucT*, *cagA*, *cagα*, *cagL*, and *vacA*.

## Materials and Methods

### Okra fresh extract fresh extract from immature fruits (FE)

Immature fruits from *A. esculentus* were obtained from a local market in Münster, Germany, and identified botanically by A.H. Voucher species (IPBP25129) are retained in the archive of the authors' institute. Beginning with 200 g material, the stems were removed and the residual material was cut into pieces. The resulting amount of 173 g material was extracted with 500 mL of water using a rotary mixer (3 min). The suspension was centrifuged at 4°C for 30 min at 14.500×*g*. The viscous extract was dialyzed (MCWO 3.5 kDa, cellulose membranes, Roth, Germany) for 3 days at 8°C. The resulting Okra FE was aliquoted into 2 mL tubes and kept at −80°C. One milliliter of Okra FE corresponds to 2.7 mg dry weight after lyophilization.

### Materials

3′-Sialyllactose (NeuAcα_2-3_Galβ_1-4_Glc), fluoresceinisothiocyanate isomer I (FITC), chloramine T, and sodium bisulfite were purchased from Sigma Chemicals (St. Louis, MO, USA). The total fraction of acidic human milk oligosaccharides [29] was obtained from the Danone Research Centre for Specialised Nutrition, Friedrichsdorf, Germany. Unless specified otherwise, all other chemicals and reagents were purchased from Merck (Darmstadt, Germany) in analytical quality.

### Bacteria and growth conditions

*Helicobacter pylori* ATCC 700824 (strain J99, identification for quality control by PCR for vacA and cacA genes) was cultivated for two or three passages to minimize the risk of phase-variable switching of OMP genes. Cultivation was performed according to [9]. We used strains CCUG17874; CCUG17875/Le^b^ [24]; CCUG17875*babA1A2*, a babA1::kan babA2::cam mutant of CCUG17875 [25]; J99*babA*, a J99babA::cam mutant of J99 [25]; J166 [36]; J166*babA* (J166*babA*::rpsl CAT CR); and strain P314, an isolate from San Juan de Miraflores, Lima, Peru [27], in addition to an *E. coli* control strain (XL10-Gold, Strategene, Santa Clara, CA, USA). Bacteria were grown on Brucella agar supplemented with 10% bovine blood and 1% IsoVitox Enrichment (Svenska LABFAB, Ljusne, Sweden) for 36–48 h at 37°C, under 10% CO_2_ and 5% O_2_ before harvest in phosphate-buffered saline (PBS) containing 0.05% Tween 20 and 1% bovine serum albumin (BSA; Saveen Werner AB, Sweden).

Two clinical *H. pylori* isolates (HP-FR1 and HP-FR2) used in this study were recovered from patients who suffered from mild antrum and corpus gastritis. Patients' gastric tissue samples were cultured on agar media containing 10% (v/v) washed human erythrocytes and 10% (v/v) heat inactivated horse serum with (HHPZ) and without (HHP) antibiotic supplement under microaerobic conditions for five days at 37°C; grown bacteria were identified as *H. pylori* by typical morphology of colonies, biochemical reactions (oxidase, katalase and urease) and microscopy.

### Labelling of bacteria, *Helicobacter pylori* adhesion assays, hemagglutination assay, dot blot overlay assay, gene expression analysis

The respective assays were performed as described by [6]. Primer sequences for gene expression analysis for *H.pylori* are given in Table 1.

**Table 1:** Primer sequences for *H. pylori* (Qiagen).

Primer	Gene/locus tag (Ref.)		Sequence, 5′ → 3′ orientation
23S rRNA	jhpr2 [40]	F	CCTTAGATTTACGGCGGATACA
		R	CAGTGGTAGAAGAGCGTTCATA
		P	TCACTTCATACCGCTCC
*AlpA*	jhp0848 [40]	F	GCTCACTAAAAACACCATT
		R	CATGCTTCTCACCTGATTGTTG
		P	TCCAACCAAGCTAACGC
*AlpB*	jhp0849 [40]	F	GAGTCAAAACATCAGCAAGA
		R	ACTGAGCTGGTTGGAGAGATT
		P	GACAACAACACCACGA
*BabA*	*jhp0833 * [*40*]	F	GATTTGTTATCGTTTGTCCTAC
		R	AGGCTTAGCGGGACTTTT
		P	GTTGATGG*GTTGTTGC
*HopZ*	jhp0007 [40]	F	CCAAGAAATCGTAACGCAAG
		R	TGTTTTGAGCGAAAGCCTATC
		P	TCCTTACACCTCTGCT
*OipA*	jhp0581 [40]	F	ATAAGCGAGCGTGTCAAGAA
		R	ATGCCAATCACAAGCCCTGAA
		P	GAAAGAAGG*GTAAAAGG
*HpaA*	jhp0733 [40]	F	CAAACCAGTGGAGAATAATAAC
		R	GGATAGCAGCGATAAAGACGAT
		P	CCATTCATAGCGACAG
*FucT*	jhp0596 [40]	F	TGCAAGTATCTCACGTAATCAA
		R	CTCAAGGCTATGGCTATGTAAC
		P	TGG*GAATGGTGTGGCT
*CagA*	jhp0495 [40]	F	TGGCAGTGGGTTAGTCATA
		R	CCTGTGAGTTGGTCTTCTTTGT
		P	AGGTGGTGAGAAAGG*GA
*CagL*	jhp0487 [40]	F	CTCGATTTTCAGCTTCCC
		R	TCAATCCCTTAGACCAAAAGACT
		P	ATTCCGCATTGTTGCT
*Cagα*	jhp1344 [40]	F	GAGACAAGCTCCATGAGA
		R	ACCCCCGGTTCATAAAGACT
		P	ACTTATTCTCCCACTTGC
*VacA*	jhp0819 [40]	F	AAACGACAAGAAAGAGATCAGT
		R	CCAGCAAAAGGCCCATCAA
		P	CAATAGCAACACAGAGG
*Ure I*	jhp0066 [40]	F	AGTGTTGATCGCTACGAATAAG
		R	AGCGACTGGGTTATTGTTTGG
		P	AGTGTGGTTGATAGCGG
*UreA*	jhp0068 [40]	F	TTGCCTTCGTTGATAGTGATG
		R	CTGATGGGACCAAACTCGTAA
		P	AACAACTCACCAGGAA

F  =  Forward Primer; R  =  Reverse Primer, P  =  QuantiProbe (label  =  FAM); *  =  modified nucleotide.

### Cell culture

Human adherent gastric adenocarcinoma epithelial cells (AGS, ATCC CRL-1730) were kindly provided by Prof. W. Beil (Medizinische Hochschule Hannover, Germany). Cells were grown as described previously in [9].

### Radiolabeling of Okra FE and glycoconjugates

Radiolabelling [26] of Okra FE, which contains a substantial amount of glycoproteins, was performed as described by [12] using 2 µL of Okra FE dissolved in 50 µL buffer. The same procedure was applied to Lewis^b^ and sialyl-Lewis^x^ [12].

### Analysis of binding properties of Okra FE towards different *H. pylori* strains by radioimmunoassay

^125^I-labeled fractions of Okra FE were diluted 10-fold with blocking buffer (PBS containing 1% BSA and 0.05% Tween 20). The tested strains were *H. pylori* strains J99, J99*babA*, J166, J166*babA*, CCUG17874 (17874), CCUG17875/Le^b^ (17875/Le^b^), CCUG17875*babA1A2* (17875*babA1A2*), and P314; and *E. coli* XL10-Gold. A bacterial suspension adjusted to OD_600_ 0.1 per mL was used. Ten microliters of radiolabeled sample were added to 1 mL of bacterial suspension. After mixing, samples were incubated for 17 h at RT on a rocking table. Bacteria were pelleted by centrifugation (16,058×*g*, 13 min) and the supernatant was separated from the pellet. Both supernatant and pellet were collected into plastic tubes and measured with a gamma counter (counts per min) for 10 min. Differences in radioactivity between the pellet and the supernatant reflect the ability of bacteria to bind to the test compounds.

### Inhibitory effects of FE on Lewis^b^ binding of *H. pylori*

An OD_600_ 0.1 suspension of two different bacterial strains (J166, P314) was prepared in citrate buffer, pH 5. Five hundred microliters of each suspension was transferred to reaction tubes. For the third tested strain, 17875/Le^b^, the suspension was diluted to an OD_600_ of 1.3×10^−4^ by mixing with *H. pylori* strain 17874 (OD_600_ 0.1/mL) and blocking buffer (PBS with 1% BSA (Cohn fraction V, protease-free, Saveen & Werner AB, Limhamn, Sweden)). Addition of 17874, a strain that does not bind Le^b^ [32] was necessary to create a visible pellet.

For the CCUG17875/Leb strain, which has a high binding affinity (K 3.9E+11 M^−1^) [27], the bacterial suspension was serially diluted to an OD 600 of 1.3×10^−4^ in order to meet an assumption of an analysis of binding experiment that only a very small fraction of the radioligand binds both specifically and non-specifically [33].

Next, 450 µL of sample solutions with different concentrations were added to the reaction tubes. Finally, 50 µL (0.2 ng) of radiolabeled Le^b^-HSA was pipetted carefully into each reaction tube. Samples with bacteria and radiolabeled Le^b^-HSA alone served as controls (no inhibitor added), and samples with radiolabeled Le^b^-HSA and *H. pylori* strain 17874, which does not bind Le^b^, served as a blank. After mixing, samples were transferred to a rocking table and were incubated and measured.

### Inhibitory effects of FE on sialyl-Lewis^x^ binding of *H. pylori*

These experiments were performed similarly to those described above regarding Le^b^ binding; however, blocking buffer, which contains periodate-treated BSA that lacks sialic acid, was used instead of citrate-phosphate buffer. The use of this pre-treated BSA is necessary during experiments concerning the SabA adhesin of *H. pylori* because the sialic acid structures in BSA may interfere with the adhesin, leading to false results [27]. *H. pylori* strain 17875*babA1A2* was used to determine SabA-mediated binding, and 17875/Le^b^ served as a negative control. An amount of 1 ng/mL iodinated sLe^x^ was used for these experiments.

### Adhesion of *H. pylori* to magnetic nanoparticles (MNP)

Magnetic nanoparticles with various surface modifications and coatings were used to investigate the influence of selected polysaccharides and particle charges on adhesion to *H. pylori*. FluidMAG®-ARA, fluidMAG®-DX, fluidMAG®-UC/A, and fluidMAG®-UC/C MNPs (Chemicell, Berlin, Germany) (specification 200 nm size, 2.2×10^14^ particles/g, 25 mg/mL weight to volume for MAG-ARA and MAG-DX, and 35 mg/mL for MAG-UC/A and MAG-UC/C) were diluted to 0.5 mg/mL in sterile PBS. Agar-grown *H. pylori* strain J99 (48 h) was harvested and suspended in PBS, and 7 mL of a bacterial suspension with an OD_550_ of 2.0 was prepared. One milliliter of the bacterial suspension was mixed with 1 mL of the MNP suspension. For controls, every MNP sample was prepared in the same way with PBS instead of bacterial suspension. Samples containing bacteria alone (OD_550_ 1.0) were prepared to serve as untreated controls. Suspensions were incubated for 1 h at 37°C with standardized shaking intensity. After this incubation step, reaction tubes were fixed on external magnets and left for 20 min at RT. Control samples with no MNPs were also exposed to a magnetic field. As MNPs were forced by the magnetic field to attach to the wall of the reaction tube, an MNP-free supernatant was formed. One milliliter of this MNP-free supernatant was removed and placed in a plastic cuvette. The OD_550_ of the supernatant from every sample was measured. The OD_550_ of bacteria-free samples served as blanks and were subtracted from the OD_550_ of respective bacterial samples. The OD_550_ of the untreated control was calculated as 100%, and sample ODs were related to this value.

### Statistics

Statistical results were obtained by the use of SPSS® statistics software volume 20 (IBM, Armonk, USA). Results were expressed as mean value (MV) ± standard deviation (SD). After Levene's test on variance homogeneity, analysis was performed using one-way analysis of variance (one-way ANOVA). If results revealed significant differences between group mean values, then groups were compared using the Dunnett test (2-sided), with *p*<0.05 considered statistically significant (*) and *p*<0.01 considered highly statistically significant (**).

## Results

### Antiadhesive activity of Okra FE against *H. pylori*

**Figure 1:**
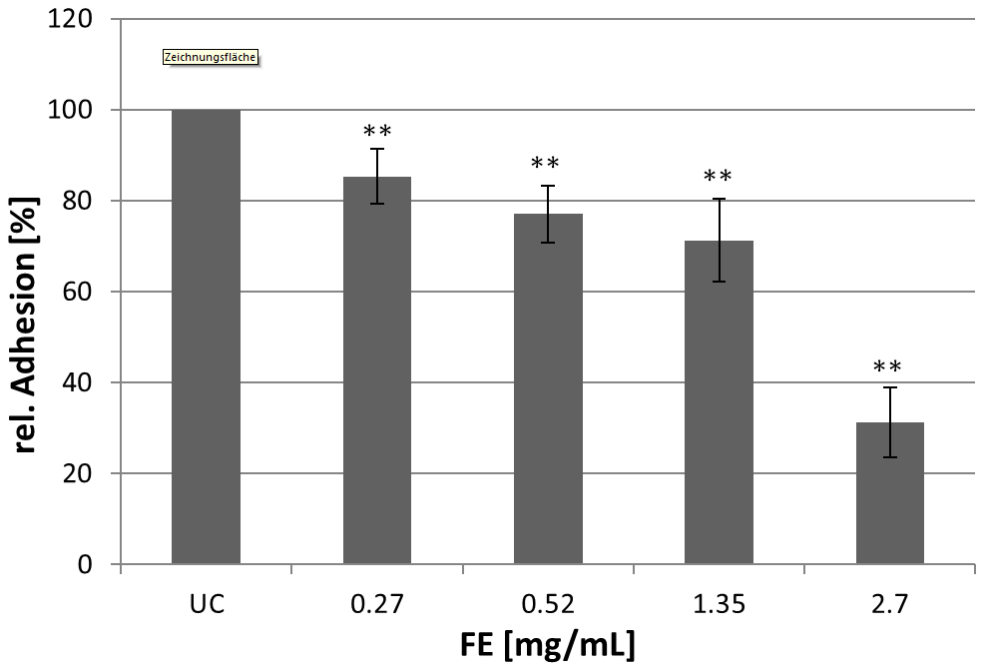
Relative adhesion (%, related to the untreated control UC) of FITC-labeled *H. pylori* to AGS cells after pretreatment of the bacteria with different concentrations of Okra FE. UC: untreated control; Values are mean ± SD; n = 3 independent experiments with 3 replicates each, ** p<0.01.

Aqueous Okra FE was prepared by macerating the immature fruits in water under standardized conditions. The suspension was centrifuged, followed by dialysis at 8°C. Then the resulting Okra FE was aliquoted and stored frozen at −20°C. Its respective dry mass was 2.7 mg/mL. Lengsfeld et al. [3] provides an analytical characterization of the glycoconjugates in Okra FE, and investigation of its antiadhesive activity against *H. pylori* in a qualitative *in situ* adhesion assay system has been described in this report. An *in vitro* flow cytometric assay with human gastric epithelial AGS cells and FITC-labeled bacteria was used to quantify antiadhesive effects, as well as for mechanistic studies [5]. Okra FE exhibited a dose-dependent inhibition of bacterial adhesion to the host cells, ranging from approximately 20% to 70% inhibition in the concentration range from 270 µg/mL to 2.7 mg/mL (Fig. 1). No inhibitory activity was observed during the pretreatment of AGS cells, which indicates that Okra FE should target only the bacterial cell surface. These data are in accordance with the recently reported inhibition rates in the human gastric tissue *in situ* assay [3]. The absence of cytotoxic effects of Okra FE against *H. pylori* was verified by an agar diffusion test, and no toxic effects were detected; in addition, no cytotoxic effects against AGS cells were recorded (data not shown).

Antiadhesive activity of FE was also tested against two clinical isolates 26313 and 26322. Preincubation of strain 26313 for 2 h with FE resulted in a total bacterial adhesion of 1.3±0.6% to AGS cells, referred to the adhesion of the untreated control ( = 100%) which means about 98% inhibition. Similar data were obtained for strain 26322, which showed 18.7±2.9% adhesion, corresponding to about 82.3% of inhibition. These data are comparable to the antiadhesive properties of FE against strain J99.

In case Okra extract FE was incubated for 1.5 h at pH 1.0, which simulats gastric conditions, followed by neutralization to pH 7.0, still significant inhibition of bacterial adhesion to AGS cells was determined. While native FE gave about 90% inhibition of bbacterial adhesion (strain J99) acid-treated FE (2 h) exerted about 55±5% inhibition. Therefore, acidic conditions will decrease inhibitory activity, but significant activity is still observable.

### Okra FE interferes with BabA, SabA, and fibronectin-binding adhesins

A semiquantitative dot blot overlay assay [6] was performed to pinpoint the respective bacterial adhesins blocked by Okra FE. Therefore, putative ligands known to interact specifically with *H. pylori* adhesins were immobilized by spotting on PVDF membranes. A representative selection of ligands identified for *H. pylori* adhesins used for these experiments were: Le^b^- and H type I-conjugates (which interact specifically with BabA); sialyl-Lewis^a^, and laminin (known for interacting with SabA); and fibronectin (with a not-yet-determined bacterial adhesin affinity). In addition to the use of human serum albumin (HSA) and bovine serum albumin (BSA) as controls to exclude non-specific binding of *H. pylori* to spotted compounds on the membrane, 6′-sialyllactose also served as a control.

**Figure 2:**
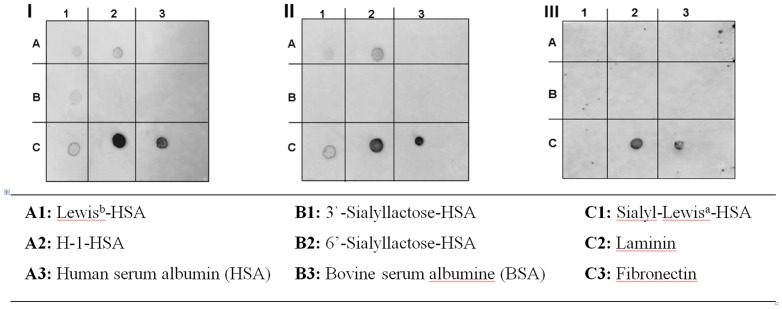
Representative images of the adhesion of FITC-labeled *H. pylori* wild-type strain J99 to immobilized ligands on PVDF membranes. (**I**) untreated control and pretreated bacteria with (**II**) 3′-sialyllactose and (**III**) Okra FE. (Neo)glycoproteins spotted on PVDF membranes (1 µg per spot) were overlaid with FITC-labeled *H. pylori* and adherent bacteria were detected by fluorescence imaging. The respective locations of spotted (neo)glycoproteins are indicated below.

As expected, significant bacterial adhesion of untreated *H. pylori* to the immobilized ligands was obvious (Fig. 2I). No adhesion to spotted 3′-sialyllactose-HSA conjugate was observed when bacteria were preincubated with 3′-sialyllactose (Fig. 2II). Pretreatment of *H. pylori* with Okra FE blocked binding to the respective immobilized HSA-conjugates of Le^b^, H-1, 3-sialylactose, and sialyl-Lewis^a^, and also strongly reduced binding to spotted laminin and fibronectin (Fig. 2III). Therefore, it can be deduced that Okra FE interacts with BabA, SabA, and an unknown fibronectin-binding adhesin. This multi-targeted, focused inhibition also explains the strong reduction of *H. pylori* adhesion to gastric epithelial cells in *in vitro* and *in situ* conditions.

### FE interacts with SabA and inhibits *H. pylori*-mediated hemagglutination

A hemagglutination assay using human blood (0-) was performed in order to prove the interaction of FE with SabA, the hemagglutinin of *H. pylori* with strong binding affinity to NeuAc2α2-3Gal [16]. Acidic human milk oligosaccharides served as a positive control, because they contain structures of α-2,3-linked sialic acid motifs [29] together with fucosylated oligosaccharides and should reduce *H. pylori*-mediated hemagglutination [28]. Acidic milk oligosaccharides revealed a reduction of agglutination titer within 1∶2 serial dilution assays of 2.5+/−0.8 titer steps at 1 mg/mL test concentration. Okra FE at 2.2, 1, and 0.1 mg/mL test concentrations reduced the titer of hemagglutination by 3, 3, and 0.5 titer steps (2 independent experiments with 2 replicates each), respectively. These data indicate an interaction of Okra FE compounds with SabA.

### Binding of radiolabeled Okra FE to different strains

**Figure 3:**
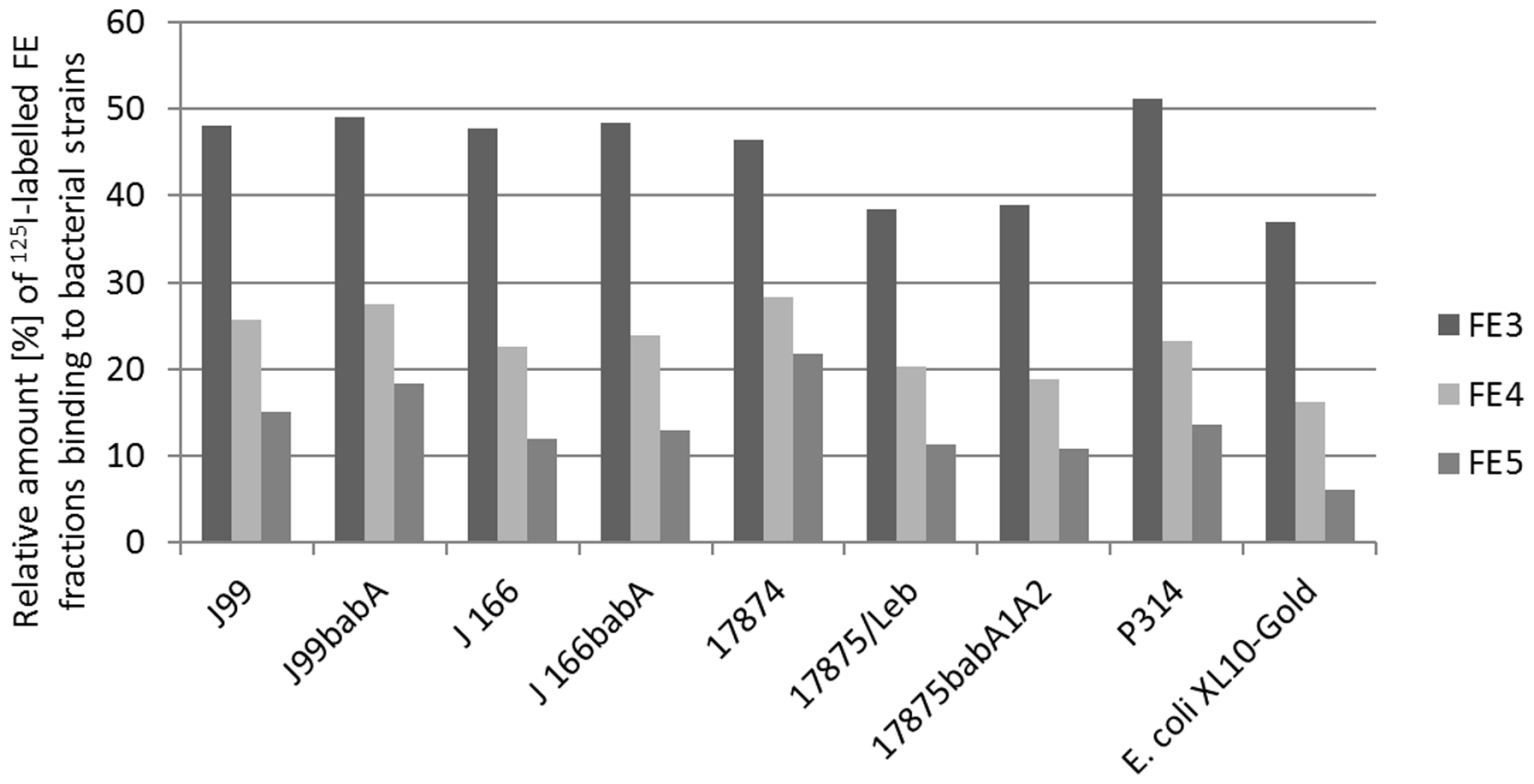
Binding of radiolabeled fractions Okra FE subfractions FE3, 4 and 5 to different *H. pylori* bacterial strains and *E. coli* reference strain.

Radioimmunoassays were performed to analyze whether the reduced binding of *H. pylori* to H-1 and Le^b^ after the bacteria is pretreated with Okra FE is mediated by specific interaction with BabA, and to prove that SabA binding to sLe^x^ is inhibited. Peptide-bearing polymers from Okra FE were radiolabeled [26], purified, and fractionated by PD-10 gel permeation. The maximal radioactivity was obtained for three fractions (FE3, FE4, and FE5) representing the radiolabelled high molecular glycoproteins. Le^b^-HSA and sLe^x^-HSA were also iodinated in a similar way. Binding experiments against a variety of different *H. pylori* strains revealed strong interactions of FE3, FE4, and FE5 with all strains. Although FE3 (the highest molecular weight fraction) exhibited the strongest interaction, FE4 and FE5 also interacted with the bacteria (Fig 3). In principle, similar Okra FE binding was observed for all strains tested (e.g., for the wild type strain J99 (BabA, SabA positive), as well as for the J99 BabA deletion mutant). The same was true for wild type J166 (BabA, SabA positive) and its respective BabA mutant. Strain 17874 (BabA neagtiv, SabA positive), strain 17875/Le^b^ (BabA positive, SabA negative), BabA deletion mutant 17875*babA1A2* (BabA negative, SabA positive), and strain P314 (BabA positive) also interacted with FE fractions. Therefore no specifity for BabA interaction can be seen as no difference between the binding to strains J99 and J166 and to the respective *babA* deletion mutants was observed. Moreover, the interaction of FE does not appear to be specific for *H. pylori*, because the labeled bacteria also bound to the *E. coli* control strain.

### Competiton of Okra FE with Le^b^ and sLe^x^ ligands

**Figure 4:**
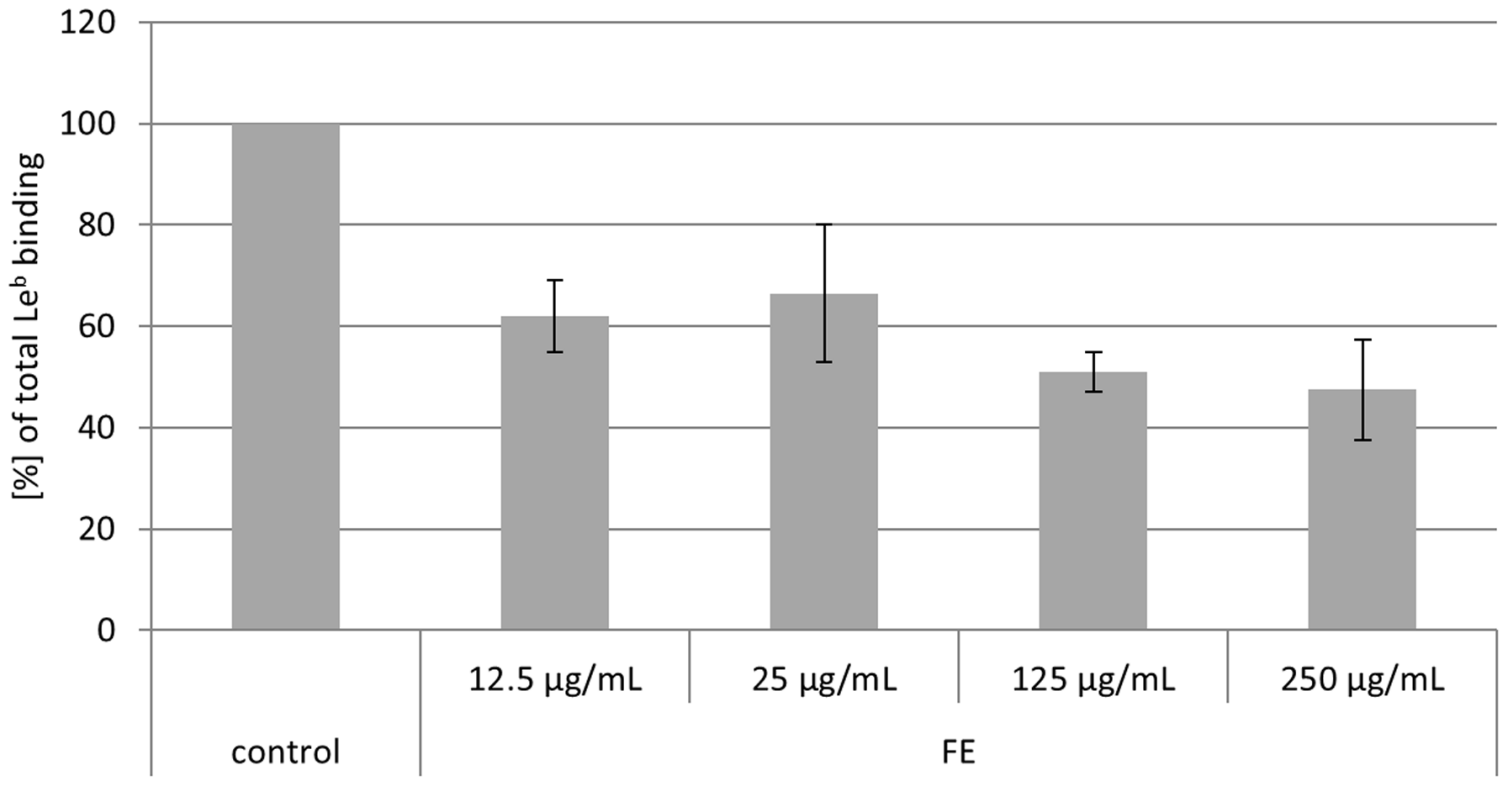
Influence of Okra FE on *H. pylori* strain 17875/Le^b^ binding to Le^b^-HSA. Data are related to the Le^b^-HSA binding of the control (only 17875/Le^b^ together with Le^b^,  = 100% Le^b^ binding).

To investigate whether Okra FE influences the binding of *H. pylori* to Le^b^, we coincubated labeled Le^b^-HSA conjugate with unlabeled Okra FE in the presence of the strong Le^b^ binding strain 17875/Le^b^ (Fig. 4). FE strongly inhibited the binding of 17875/Le^b^ to Le^b^-HSA. Inhibitory effects of approximately 25 to 60% were achieved in the concentration range of 12.5 to 250 µg/mL, supporting earlier observations of high antiadhesive activity [3].

To see whether these effects also occur with the *H. pylori* strains J166 and P314, which are known to have an approximatly 100-fold lower affinity for Le^b^ compared with 17875/Le^b^ (J166: *K*_a_ 4.34E+09 M^−1^; P314: *K*_a_ 5.57E+09 M^−1^) [27], we also tested these strains. Concentrations of Okra FE up to 500 µg/mL did not reduce the binding of Le^b^ to strain P314 (data not shown). In contrast, a clear, but low level of binding inhibition was observed in the concentration range of 12.5 to 500 µg/mL for the J166 strain. At a concentration of 500 µg/mL, Le^b^ binding was inhibited to approximately 30% of the control level. This was an unexpected finding, because although the binding of Okra FE occurs in all strains tested (Fig. 3), this binding is not necessarily related to the inhibition of BabA; again, this binding appears to be strain-specific. Given this result, it might be assumed that the interaction is not at the active site of the OMPs, but is caused by a more nonspecific interaction with the bacterial surface in the vicinity of the adhesins, which in turn decreased the functionality of the OMPs, depending on the respective surface organization.

**Figure 5:**
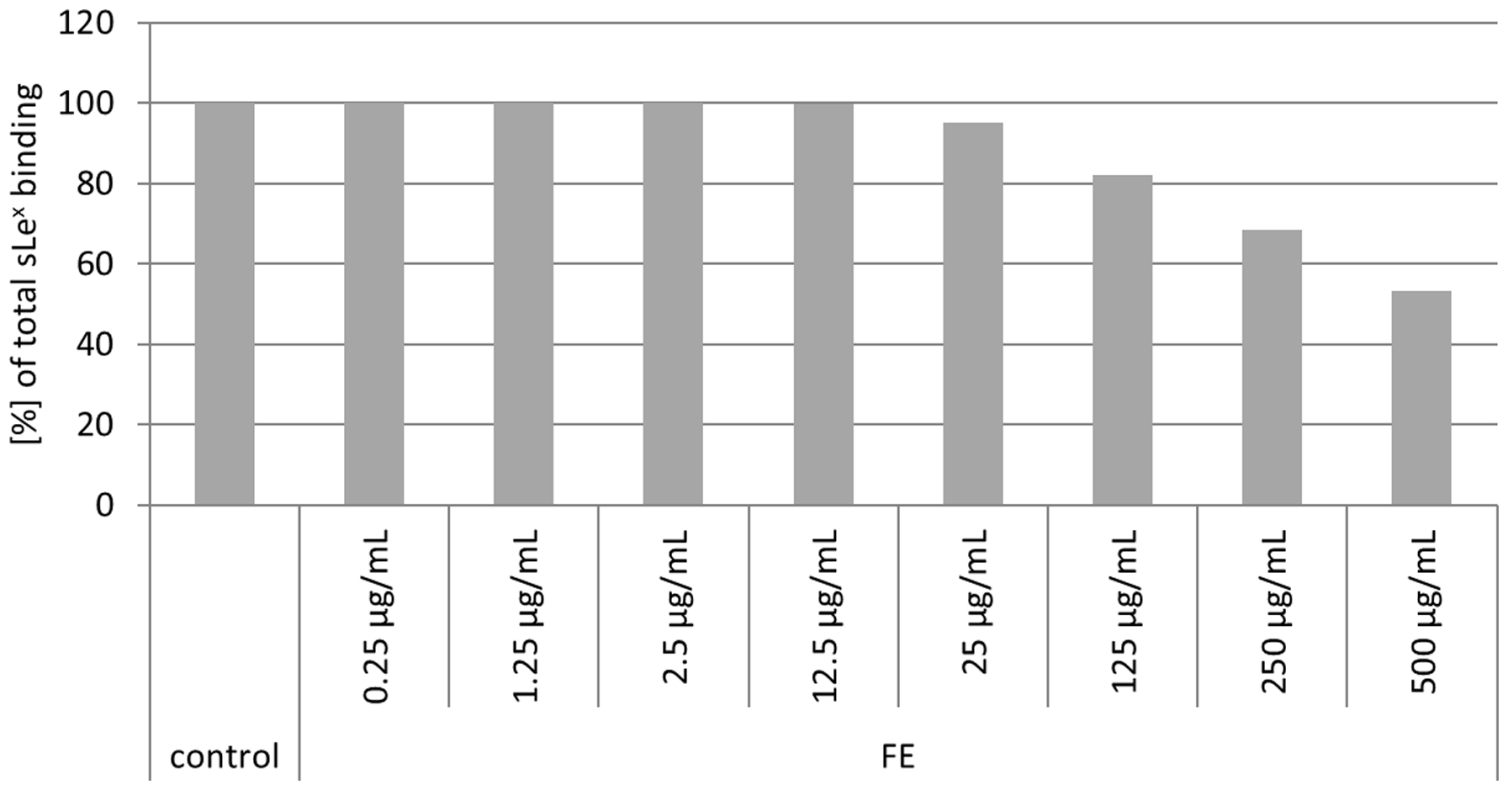
Influence of Okra FE on *H. pylori* strain 17875*babA1A2* binding to sialyl-Le^x^-HSA. Data are related to the control (only 17875*babA1A2* with sLe^x^-HAS  =  100%).

To investigate the influence of Okra FE on *H. pylori* binding to sialyl-Lewis^x^, radioimmunoassay investigations were performed using strain 17875*babA1A2* because it binds sLe^x^, but not Le^b^ [25]. The 17875/Le^b^ strain served as blank, because it does not bind sLe^x^ [25]. The results of these experiments are shown in Fig. 5. The average binding percentage of sLe^x^-HSA to 17875*babA1A2* was 36% for the controls of the performed tests. FE exhibited a strong influence against strain 17875*babA1A2*, binding sLe^x^ at a concentration range of 25 to 500 µg/mL with 5% to 47% dose-dependent inhibition of bacterial binding. This finding suggests inhibition of SabA-mediated adhesion.

### Binding of polysaccharides to *H. pylori* depends mainly on the molecular charge

**Figure 6:**
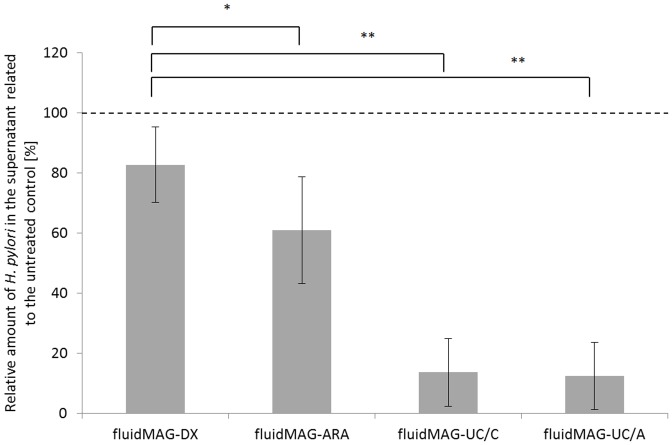
Adhesion assay with MNPs. Amount of bacteria in the untreated control was calculated as 100% and is indicated as broken line. Results of the samples incubated with MNPs are related to the values of the untreated control. Samples are named according to the type of MNPs added. Results are mean values with ± SD from six independent experiments with *: *p*<0.05 and **: *p*<0.01. Significance values refer to the comparison with the fluidMAG-DX® values, as indicated by the brackets.

Based on these results, the binding of polymers from FE appears to be more or less nonspecific, but effectively leads to an inhibition of bacterial adhesion. We used commercially available magnetic nanoparticles (fluidMAG®) coated with the neutral polysaccharide dextran (fluidMAG-DX®) to investigate whether polysaccharides generally bind to the *H. pylori* surface. An *H. pylori* suspension was incubated under standardized conditions with the nanoparticles, and then bacteria with nanoparticles stuck to the cell surface were captured by an external magnetic field. The relative amount of *H. pylori* in the incubation medium, not fixed to the magnet, was determined by OD measurement. As shown in Fig. 6, nearly all bacteria (>84%) were present in the medium, which means that the dextran-coated nanoparticle is not interacting with the bacterial surface. This finding indicates that neutral polymers do not bind to *H. pylori*. Magnetic nanoparticles coated with polyarabinic acid (fluidMAG-ARA) were also used in the same experiment to represent a strongly acidic polysaccharide with terminal glucuronic acid residue. This construct had a significantly higher binding affinity to the bacteria than the dextran-coated particles (Fig. 6), which indicates that molecular charge likely contributes significantly to the interaction with the bacterial surface. Uncoated particles were used to investigate whether the carbohydrate moiety also contributes to binding affinity; however, these MNPs bore a cationic or an anionic charge (fluidMAG-UC/C and fluidMAG-UC/A). Surprisingly, both nanoparticles exhibited a very strong affinity for the bacteria, resulting in approximately 90% reduction of bacterial titer in the incubation medium (Fig. 6). Therefore, we assume that the interaction of large molecules with the *H. pylori* surface depends mainly on the charge of the polymer.

### Differential gene expression

To determine the influence of Okra FE on the gene expression of bacterial adhesins and virulence factors, and especially on the potential correlation of adhesion with the expression of virulence factors, *H. pylori* was incubated with FE. Using 23S rRNA as an endogenous control, we monitored the influence of Okra FE on several OMPs (*babA*, *alpA*, *alpB*, *hopZ*, *oipA*, and *hpaA*). Moreover, the gene encoding *α-*1,3-fucosyltransferase (*fucT*), which is involved in catalysis of the Lewis^x^ trisaccharide (a major component of *H. pylori* lipopolysaccharides), was also included in the study [30]. We also assessed genes encoding *vacA* and *cagA*, as well as *cagL* and *cagα* (encoded in the cagPAI pathogenicity island for the type 4 secretion system TFSS), the metalloenzyme urease (*ureA*), and a regulator for the transport of urea by an acid-gated urea-channel (*ureI*) [31].

**Figure 7:**
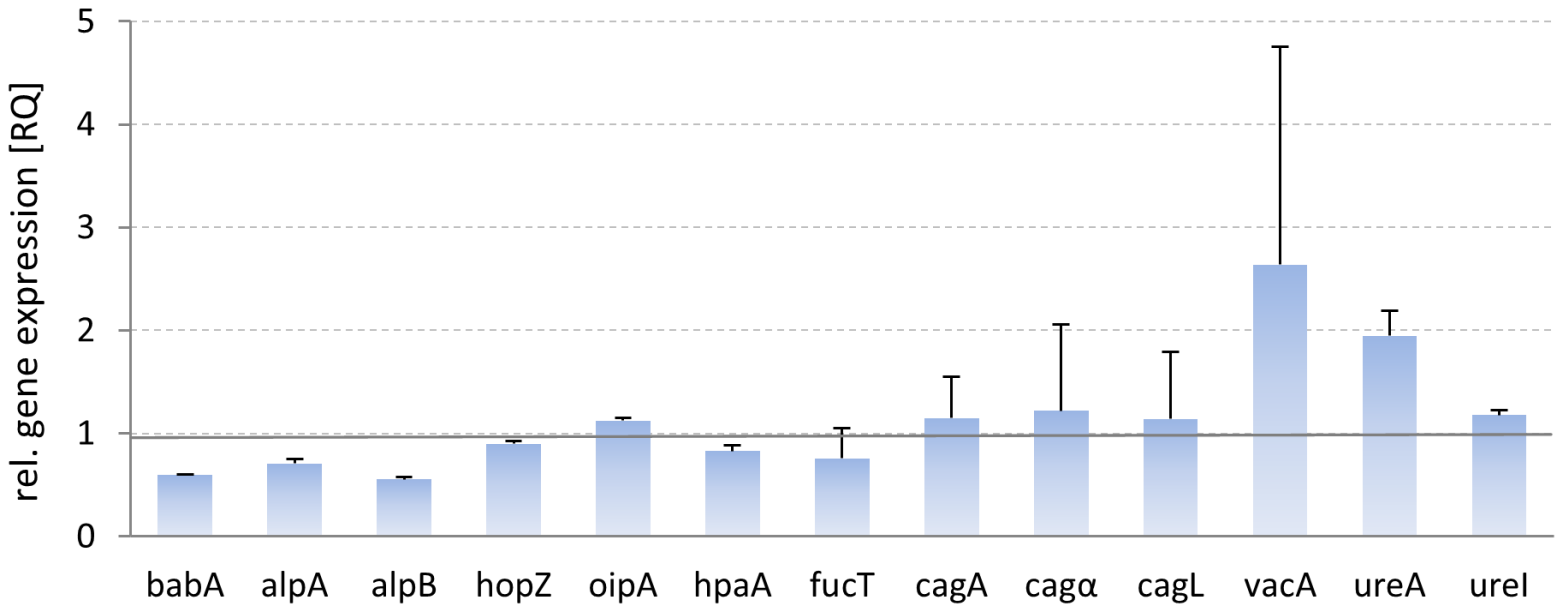
Differential gene expression of *H. pylori* pretreated with Okra FE. Endogenous control: 23S rRNA. Data are related to untreated control (UC) *H. pylori* in liquid growth medium (RQ = 1), with n = 2 replicates from three independent experiments (mean ± SD). * *p*≤0.05

For this study, the gene expression of untreated *H. pylori* was set as the reference (RQ = 1) in relation to the gene expression of pretreated bacteria. Preincubation with FE resulted in the small but significant downregulation of three OMP genes: BabA (RQ = 0.60), AlpA (RQ = 0.71), and AlpB (RQ = 0.55). The expression of genes encoding all virulence factors did not change significantly, with vacA and ureA showing a tendency toward increase (Fig. 7). These data indicate that the interaction of FE with the adhesins does not induce an automatic feedback mechanism in the bacterial cell that is associated with increased or dramatically decreased functionality.

## Discussion

Although eradication therapy of *H. pylori* has been successfully implemented, considerably high recurrences rates are documented. The annual reinfection rate is quite low in developed countries (3%), but considerably higher in developing countries (13%) [38]–[39]. In addition, the increasing antibiotic resistance of *H. pylori* is being monitored. Therefore, advanced molecular targets must be pinpointed for future *H. pylori* treatment. The development of antiadhesive compounds that interfere with OMPs and block bacterial adhesion might be an interesting approach for prevention [34]. Most *H. pylori* infections occur during the first 2 to 5 years of life [35]; in principle, the development of such antiadhesive compounds toward products for use in food or health products might help to prevent very early infection in children [6]. After antibiotic treatment, some patients experience recurrence of the infection after several months (a problem mainly in developing countries, such as South America and Asia), and it is possible that these patients might benefit from the use of such compounds in food supplements to be used during and after antiobiotic eradication therapy. In this context, translational developments of antiadhesive drugs such as Okra extract (which is already used in traditional Indian medicine for gastritis) are of interest.

The chemical synthesis and optimization of specific inhibitors of the major adhesins BabA and SabA might be possible, and might be a promising tool for future pharmaceutical and clinical development. For optimized *in silico* definition and chemical synthesis of inhibitors with specific activity toward the active center of these lectin-like proteins, more detailed investigations regarding the molecular and physical characteristics of the adhesins are necessary; specifically, no protein crystal data have been published at this time.

Such specific BabA inhibitors with an underlying structure-activity relationship have recently been described for the class of N-phenyl-propenoyl-amino acid amides [30]; however, specific blocking of BabA only leads to a 20% to 30% reduction of bacterial adhesion. Because other adhesins (SabA, Alp, OipA, HopZ, etc.) are sufficient to achieve successful adhesion, the inhibition of BabA alone does not strongly minimize infection of the stomach cells. This finding may trigger a paradigm shift from the use of a single, highly specific inhibition molecule to the development of “multi-target oriented approaches” with compound mixtures that interact with different OMPs, which will lead to a more pronounced inhibition of bacterial adhesion. We used this strategy in the above described experiments by employing a crude polysaccharide- and glycoprotein-containing extract from immature okra fruits that reduces bacterial binding much better than has been described for other inhibitors, such as decapeptides [6], 3-sialyllactose [5], or N-phenylpropenoylaminoacid amides (NPAs) [9].

Questions might arise regarding the possible influence of such adhesion blockers as Okra FE on the intestinal flora, particularly whether they also influence the adhesion of colonic bacteria, which could theoretically lead to a decrease in bacterial density and result in clinical symptoms such as diarrhea. We assume that this will be not a great problem for Okra FE, because okra is eaten widely throughout Asia and Africa, and no undesired effects on the gastrointestinal system have been reported. In addition, animal experiments regarding Okra FE use in chickens have not indicated any negative influence [20].

Another problem has to be discussed, namely the phenomenon that under *in vivo* conditions most *H. pylori* will be located in or on the gastric mucus phase. From previous investigations [unpublished data] we know that okra polysaccharides have no affinity to rat stomach epithelia or mucilaginous effects to intact tissue; this has been proven within an ex vivo adhesion bioassay. For that we have the feeling that FE will only interact with free floating bacteria in the stomach liquid, or bacteria at a very early stage after entry into the organism.

It is interesting that Okra FE does not interact directly with the active sites of BabA or SabA, but with still-unknown surface structures in the vicinity of the adhesins. This means that the nonspecific inhibiting effects of Okra FE may not be affected by the mutation of the adhesins. Therefore, a discussion of the possible future developments of active pharmaceuticals with a low degree of specifity must be considered. Going back into the early 1940s and 1950s, the pharmaceutical industry has developed many somewhat nonspecific antibiotics: β-lactam antibiotics are definitely not as specific as we would like, and are capable of interacting with entire families of penicillin-binding proteins. Furthermore, inhibitors of protein translation interact with more than one ribosomal protein. Regarding other classes of widely used drugs, even aspirin and paracetamol are valuable active ingredients not because they are extremely specific, but because they affect a very broad range of molecular targets.

It must be kept in mind that antiadhesive compounds such as the Okra FE would likely have to be used as lifelong preventive or therapeutic treatment, and undesired effects on intestinal flora and the intestine might occur, especially due the low degree of specifity. Future clinical developments must indicate whether the positive effects of Okra FE against *H. pylori* recurrence are more pronounced than potential side effects.

The use of crude plant extracts may be a valuable tool for future developments, also keeping in mind how economical it can be to obtain such preparations. However, immense projects must be initiated to clearly define which compound from these multitarget mixtures is directed against which OMP, and these will require an intense phytochemical and pharmacological approach for understanding such complex systems.

It has to be realized that quite high doeses of FE had to be used for inhibition of bacterial adhesion. This is in accordance to literature published on other antiadhesive candidates against *H. pylori*. For example the clinical testing of sialyllactose against *H. pylori* [5] in humans was performed with gram-doses. From the economical point of view, this makes no sense. In contrast to that the okra fresh extract can be produced easily from the plant material without big economical impact. For research we used a dialyzed extract, but for a pragmatical aspect we can think also of a very simple aqueous extract without great clean-up. For *in vivo* animal studies bigger amounts of FE have been realized recently [20]. So we have the feeling that an effective drug development should be possible.

The data presented here suggest sufficient *in vitro* antiadhesive activity, which must be proven in future animal and clinical studies. Nevertheless, the above documented results demonstrate that antiadhesive compounds like Okra FE do not enhance *H. pylori* virulence at the mRNA level. Once again, these data enhance the potential of antiadhesive compounds for future applications for the prophylactic control of *H. pylori* infections within a cytoprotective strategy. However, due to the genetic heterogeneity of *H. pylori* strains and the resulting complexity of the adhesion mechanism, further investigations with additional clinical isolates are suggested.

Okra FE can be assessed as follows: the compounds are easily manufactured by simple fruit extraction; galenical formulation of the hydrophilic compounds should not result in big problems; the stability of resultant drug compounds and of the formulation must be ensured by additional technological arrangements; the compounds must be administered in high doses (in the mg range) to affect bacterial adhesion; the compounds should not be bioavailable in the systemic compartment because of their high molecular weight and hydrophilicity; and toxicity against human cells is not expected [1]–[2]. Infection studies must evaluate whether the inhibition of a single adhesin can effectively prevent bacterial adhesion and lead to reduced infection rates.
